# Endothelial cells do not arise from tumor-initiating cells in human hepatocellular carcinoma

**DOI:** 10.1186/1471-2407-13-485

**Published:** 2013-10-20

**Authors:** Anand Ghanekar, Sharif Ahmed, Kui Chen, Oyedele Adeyi

**Affiliations:** 1 Toronto General Research Institute, University Health Network, Toronto, ON, Canada; 2 Department of Surgery, University Health Network, Toronto, ON, Canada; 3 Department of Pathology, University Health Network, Toronto, ON, Canada; 4 Toronto General Hospital, University Health Network, NCSB 11C-1227, 585 University Avenue, Toronto, ON M5G 2N2, Canada

**Keywords:** Hepatocellular carcinoma, Endothelial cell, Angiogenesis, Cancer stem cell, Tumor-initiating cell, CD31, CD34, Xenograft, Liver transplantation, Recurrence

## Abstract

**Background:**

Conventional models of carcinogenesis suggest that tumors recruit blood vessel formation from normal host tissues. This concept has recently been challenged by prominent studies of glioblastoma, which suggest that intratumoral endothelial cells (ECs) may arise from cancer stem cells/tumor-initiating cells (TICs). Hepatocellular carcinoma (HCC) is a common, highly vascularized tumor with few effective therapies, against which anti-angiogenic strategies are being actively explored. TICs are felt to play a role in HCC pathobiology, but their contributions to tumor vasculature have not been studied.

**Methods:**

We examined human HCCs in settings that selected for tumor formation from functionally defined TICs, and in which the origin of intratumoral ECs from TICs as opposed to host tissues could be clearly distinguished. We generated HCC nodules in the livers of immunodeficient mice by intrasplenic injection of HCC cells from cell lines and patient specimens and studied the tumor ECs by immunohistochemistry for mouse and human markers. We then used immunohistochemistry for EC markers in combination with fluorescence in situ hybridization (FISH) for X and Y chromosomes to study the endothelium of recurrent HCC specimens resected from sex-mismatched liver allografts of patients who had undergone liver transplantation for HCC.

**Results:**

We observed that all ECs in intrahepatic human HCC xenografts expressed mouse rather than human CD31. FISH analysis of recurrent HCCs resected from patients with sex-mismatched liver allografts revealed that all CD31^+^ and CD34^+^ intratumoral ECs originated from the donor allograft rather than the tumor.

**Conclusions:**

These observations suggest that the vasculature of human HCC arises from normal host tissues rather than from TICs, supporting ongoing efforts to target angiogenesis in HCC as it is currently understood, and suggesting that the contribution of TICs to the vasculature of other cancers is disease-specific.

## Background

Conventional models of carcinogenesis posit that tumor vasculature develops in response to factors produced by neoplastic tissues (such as vascular endothelial growth factor) that elicit the sprouting of new blood vessels from surrounding pre-existing “normal” vasculature while also promoting the recruitment and differentiation of bone marrow-derived vascular progenitor cells [1]. This concept has recently been challenged by the cancer stem cell (CSC) model, which suggests that tumors are sustained by a distinct population of cancer cells with stem cell-like properties that self-renew and are also capable of generating the full repertoire of cell types found within tumors, including those contributing to tumor vasculature [2]. Most notably, two recent reports focused on glioblastoma have provided compelling evidence that intratumoral vascular endothelium arises from CSCs [3,4].

Hepatocellular carcinoma (HCC) is one of the five most common cancers worldwide, and the third most common cause of cancer death [5]. Early-stage HCC confined to the liver is amenable to potentially curative therapies such as ablation, partial hepatic resection, or liver transplantation, though disease recurrence is common [6]. Patients with more advanced disease have a median survival less than one year due to the inefficacy of chemotherapy and radiation [7]. HCC is a highly vascularized tumor, and this characteristic is exploited as a key diagnostic criterion on radiological imaging [8]. Pathways and mediators regulating angiogenesis and neovascularization are thus recognized to play an important role in the development and progression of HCC and have emerged as attractive targets in the development of new therapies against this lethal disease [9].

Several studies suggest that CSCs, functionally assayed by their operational role as tumor-initiating cells (TICs), play a role in the pathogenesis of human HCC. Many different subpopulations of cells with tumor-initiating capacity in xenograft models have been isolated from HCC cell lines and patient tissues in recent years but their relative contributions to various aspects of HCC pathobiology remain to be clearly defined or understood [10-13]. Despite important implications for the correct targeting of new therapies against tumor vessel formation in HCC, the specific question of whether HCC vasculature arises from TICs or from surrounding tissues through “conventional” models of angiogenesis remains unexplored.

In this study, we sought to determine whether intratumoral endothelial cells in human HCC arise from TICs or from the surrounding non-cancerous tissue. We studied human HCC in biologically relevant *in vivo* models chosen for their ability to propagate tumors from single cells defined by their tumor-initiating *function* rather than a specific phenotypic cell surface marker. We first studied HCC tumor nodules generated in the livers of immunodeficient mice following intrasplenic injection of human HCC cells from established cell lines as well as patient tumors. We then examined human HCC tumors that developed in sex-mismatched liver allografts of patients who had undergone liver transplantation for treatment of HCC. We demonstrate that in both settings, endothelial cells (ECs) in human HCC do not arise from TICs.

## Methods

Ethical approvals were obtained from the University Health Network Research Ethics Board (Protocol #08-0697-TE) and Animal Care Committee (Animal Use Protocol #1595). Human tissues were obtained with written consent from patients.

### Human HCC cell lines

HepG2 cells were obtained from ATCC (catalog no. HB-8065) and grown in culture according to the supplier’s instructions. Instead of fetal bovine serum, growth medium was supplemented with 15% KnockOut SR XenoFree CTS serum replacement (Invitrogen). Huh7 cells were a kind gift from Dr. Paolo Parini (Karolinska Institute, Sweden) and were grown in DMEM/F12 media (Invitrogen) supplemented with 1X GlutaMAX (Invitrogen) and 15% KnockOut SR XenoFree CTS serum replacement (Invitrogen). Cells were harvested for xenografting with Accutase (StemCell Technologies) according to the manufacturer’s instructions.

### Patient samples and preparation of tumor cell suspensions

HCC samples were obtained from patients undergoing surgery. Clinical data were obtained by chart review. Fresh tumor samples of approximately 1 cm^3^ were taken from the viable periphery of tumors within 30 minutes of resection and transferred on ice to the laboratory. Samples were sectioned for fixation in formalin, preservation in RNAlater (Invitrogen), or immediate digestion. Bulk tumor cell suspensions were prepared from patient samples by digestion of minced tumor fragments with 200 units/mL of type IV Collagenase (Sigma) and 0.8 units/mL of dispase II (Stem Cell Technologies) for 30-60 minutes at 37°C in an equal mixture of Dulbecco’s Modified Eagle Medium (DMEM) and F12 medium (both from Invitrogen), passage through a 70 μm cell strainer (BD Biosciences), centrifugation, and lysis of red blood cells using RBC lysis buffer (eBioscience). Human CD45^+^ cells and dead cells were excluded by fluorescence activated cell sorting on a FACSAria II cell sorter (BD Biosciences) after staining with anti-human CD45 PE-Cy7 (BD Biosciences) and Live/Dead Fixable Violet Dead Cell Stain Kit (Invitrogen) as per the manufacturer’s instructions; the viable CD45^-^ fraction was then used for xenografting.

### Xenografts

6-8 week old female NSG mice (non-obese diabetic severe combined immunodeficiency interleukin 2 receptor gamma chain null) (Jackson Laboratory) were anesthetized with inhalational isoflurane. Using aseptic surgical technique, a left subcostal incision was made and the spleen exposed. 10^6^ HCC cells in 30 μL of cold Matrigel (BD Biosciences) were injected into the lower pole of the spleen using a 29-gauge needle. Upon removal of the needle the lower pole of the spleen was ligated with a silk ligature and the abdominal incision then closed with sutures. After recovery from surgery, animals were observed for 90 days and then sacrificed. Tissues were fixed in formalin or preserved in RNAlater. The xenografts analyzed in this study were generated from two different human HCC cell lines (HepG2 and Huh7) as well as from primary cells isolated from three different HCC specimens originating from three distinct patients. Xenografts were generated in duplicate in different mice from each cell line or patient sample and analyzed independently as described below.

### Reverse transcription polymerase chain reaction (RT-PCR)

Ribonucleic acid (RNA) was isolated using TRIzol (Invitrogen) from tissues stored in RNAlater and then analyzed as previously described [14].

### Immunohistochemistry and in situ hybridization

Formalin-fixed, paraffin-embedded tissues were utilized. Hematoxylin and eosin (H & E) stains were performed using standard techniques. For immunohistochemistry on human HCC xenografts and mouse liver tissue, dewaxed sections were blocked with 3% hydrogen peroxide, avidin/biotin blocking kit (Vector Labs), and 10% normal serum from the secondary antibody species, then incubated at room temperature with primary antibody overnight as follows: monoclonal mouse anti-human CD31 (Dako), polyclonal PECAM-1 antibody which recognizes both mouse and human CD31 (Santa Cruz), or monoclonal mouse anti-mouse H-2K (BD Biosciences). This was followed by biotin labeled secondary antibody (Vector Labs) for 30 minutes and horseradish peroxidase-conjugated ultrastreptavidin labeling reagent (ID Labs) for 30 minutes. Color was developed with DAB solution (Dako). Sections were counterstained with Mayer’s hematoxylin, dehydrated and mounted in Permount (Fisher). For analysis of human HCC specimens resected from sex-mismatched liver allografts, fluorescent immunohistochemistry was performed first using monoclonal mouse anti-human CD34 (Dako) or polyclonal goat anti-CD31 (Santa Cruz), in combination with monoclonal mouse anti-human CD45 (Dako) followed by Cy5 conjugated goat anti-rabbit or goat-anti-mouse IgG (Jackson ImmunoResearch Laboratories) or by Cy3 conjugated goat anti-mouse CD45 antibody (Jackson) according to manufacturer’s protocols. Counterstaining was performed with DAPI (Vector Labs). Images were captured with a CV-M4 + CL progressive scan monochrome camera (JAI) on a Zeiss Imager M1 using Metasystems workstation and ISIS software programs. Coordinates for all images were recorded. Slides were then washed in PBS, immersed in 100% ethanol and air dried. Fluorescence *in situ* hybridization (FISH) for X and Y chromosomes was performed using CEPX(DXZ1) SpO and CEPY(DYZ1) SpG Alpha Satellite DNA probes (Abbott Labs) as per the manufacturer’s recommendations. Dual color probes and target DNA were co-denaturated at 75°C for 5 minutes on HyBRITE followed by overnight hybridization at 37°C. Slides were washed, air dried and counterstained with DAPI. Using recorded coordinates, fields matching immunohistochemistry images were recaptured to evaluate staining of X and Y chromosomes in cells of interest by counting signals in at least 100 non-overlapping intact cells.

## Results and discussion

**Figure 1:**
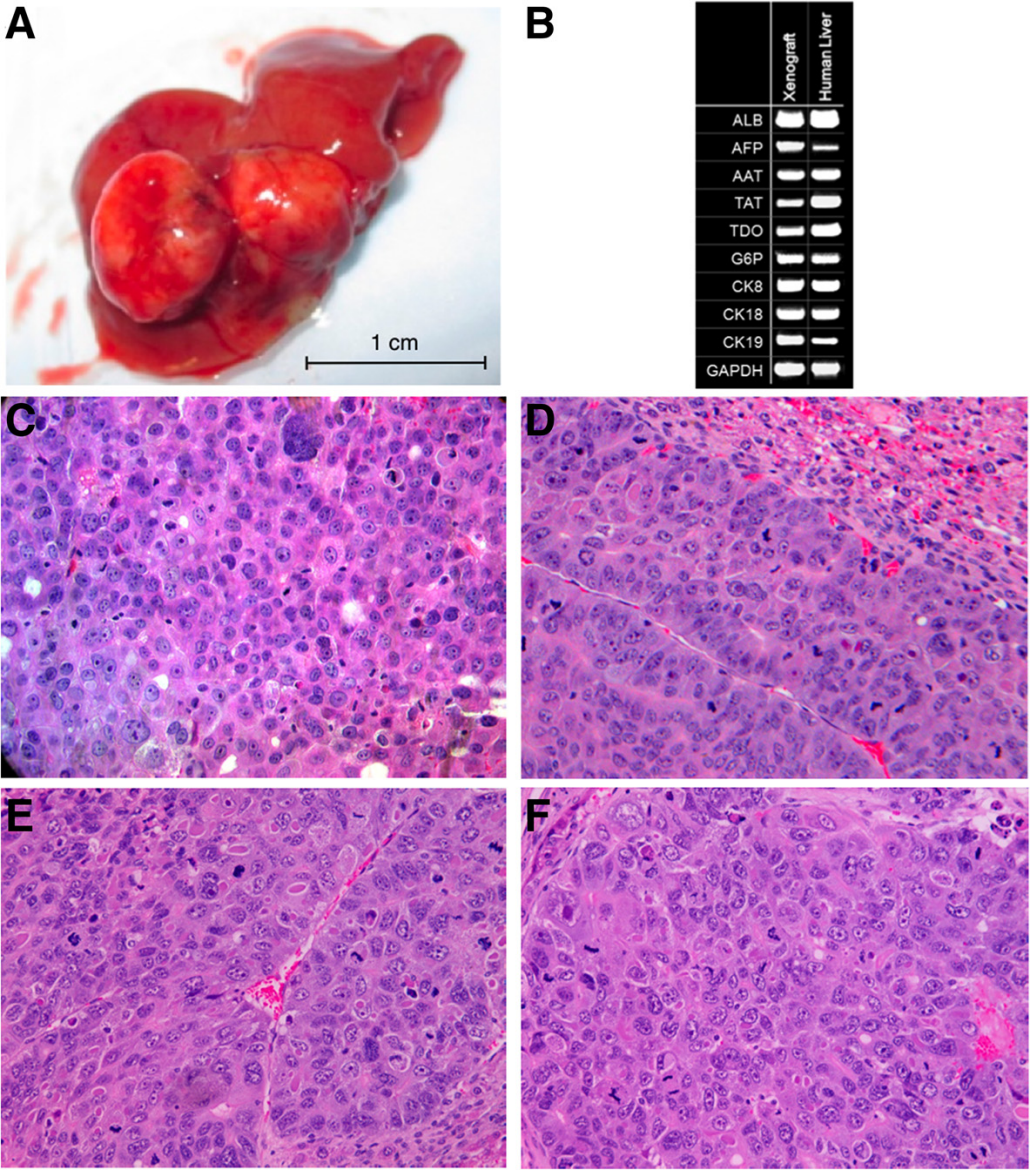
**Intrahepatic human HCC xenografts in immunodeficient mice. A)** Representative image of a murine liver with discrete human HCC nodules that formed after intrasplenic injection of primary human HCC cells isolated from a surgical resection specimen; similar nodules were also generated after injection of HepG2 and Huh7 human HCC cell lines. **B)** Representative RT-PCR analysis of a xenograft demonstrates expression of several transcripts typical of human liver tissue (composite image; ALB - albumin, AFP - alphafetoprotein, AAT - alpha-1-antitrypsin, TAT - tyrosine aminotransferase, TDO - tryptophan-2,3-dioxygenase, G6P - glucose-6-phosphate dehydrogenase, CK8/18/19 - cytokeratin 8/18/19, GAPDH - glyceraldehyde phosphate dehdrogenase). **C)** Histopathology of a resected patient tumor demonstrates typical features of HCC including hepatocyte-like cells with nuclear atypia and high nuclear:cytoplasmic ratio, absence of portal tracts, and distorted trabeculae with increased thickness of hepatocellular plates (H & E stain, x200). **D)** Section of murine liver demonstrating normal murine liver tissue (top right) adjacent to intrahepatic human HCC xenograft that closely resembles the corresponding original parent tumor shown in panel **C** (H & E stain, x200). **E)** Histopathology of intrahepatic xenograft generated from Huh7 cells (H & E stain, x200). **F)** Histopathology of intrahepatic xenograft generated from HepG2 cells (H & E stain, x200).

We focused on generating xenografts within the murine liver in order to study tumor vasculature in the microenvironment most relevant for human HCC pathobiology. By injecting human HepG2 and Huh7 HCC cell lines or primary CD45^-^ bulk HCC cells isolated from patient tumors into the spleens of recipient NSG mice, we generated intrahepatic human HCC xenografts consisting of discrete nodules (Figure 1A) that presumably originated from single tumor-initiating cells (TICs) that had migrated into the mouse liver via the splenic vein and portal circulation [15]. We analyzed these nodules by RT-PCR to confirm the expression of liver cell markers typical of HCC (Figure 1B), and found that the xenografts demonstrated histologic features typical of HCC including hepatocyte-like cells with nuclear atypia and high nuclear:cytoplasmic ratio, absence of portal tracts, and distorted trabeculae with increased thickness of hepatocellular plates (Figure 1D, 1E, and 1F). When patient-derived HCC cells were used, the xenograft histology was similar to the original patient tumor (Figure 1C and 1D). We have utilized this approach successfully with HCC samples from several patients with reproducible results [16].

Because of the likelihood that cell surface marker phenotypes of TICs vary between cell lines and among different patient tumors, we utilized the approach of intrasplenic injection of bulk tumor cells in order to select for HCC cells with tumor-initiating *function* in an unbiased manner rather than purify specific populations from the wide variety of putative HCC TICs described in the literature. By injecting cells in the spleen rather than directly into the mouse liver, we also hoped to avoid the potentially confounding contributions to xenograft vasculature from human ECs isolated along with bulk tumor cells from enzymatically digested HCC specimens. Furthermore, we believe this indirect approach yields intrahepatic tumor nodules derived from single HCC cells rather than from cell aggregates that might yield tumors from bulk tumor cell suspensions implanted directly into the mouse liver.

**Figure 2:**
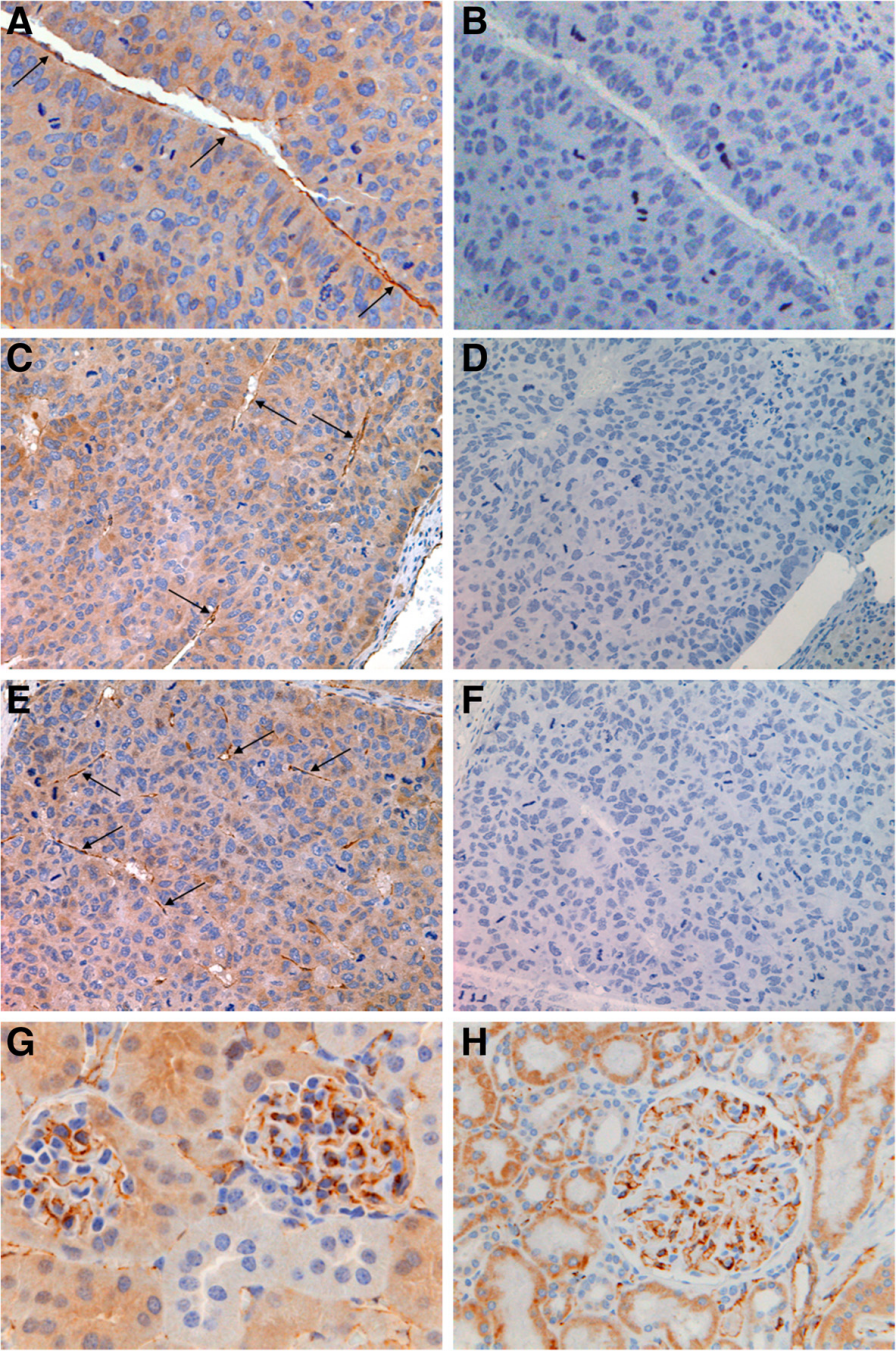
**CD31 staining demonstrates that ECs in human HCC xenografts within the mouse liver are of mouse rather than human origin. A)** Representative immunohistochemical staining with a polyclonal antibody recognizing mouse and human CD31 demonstrates endothelial cells in sinusoid-like structures (arrows) within patient-derived human HCC xenografts in the murine liver (x200). **B)** Immunohistochemical staining of the same section as in Panel **A** with a monoclonal antibody against human CD31 is negative (x200), suggesting that ECs in the xenografts are of mouse origin. Similar staining patterns with the two antibodies are observed in intrahepatic xenografts generated from Huh7 cells (Panel **C** – polyclonal anti-mouse/human CD31, and Panel **D** – monoclonal anti-human CD31) and HepG2 cells (Panel **E** – polyclonal anti-mouse/human CD31, and Panel **F** – monoclonal anti-human CD31) (all x100). **G)** Positive control staining of murine kidney with polyclonal anti-CD31 demonstrates expected pattern of positivity of the glomerular endothelial cells (x200). **H)** Positive control staining of human kidney with monoclonal antibody against human CD31 demonstrates expected pattern of positivity of glomerular ECs verifying activity of the antibody used in Panels **B**, **D**, and **F** (x200). Staining of murine kidney with the monoclonal antibody against human CD31 was negative (not shown), confirming that this antibody is specific for human CD31.

To determine whether intratumoral ECs were derived from TICs, we used immunohistochemistry to ascertain whether the vasculature of human HCC xenograft nodules in the murine liver was of mouse or human origin. Because initial staining for the mouse histocompatibility molecule H-2K clearly identified structures with vascular morphology within xenografts (not shown), we stained more specifically for murine and human CD31 (PECAM-1), an endothelial cell marker. As shown in Figure 2A, immunohistochemical staining of patient-derived HCC xenografts within the murine liver using a polyclonal antibody recognizing mouse and human CD31 demonstrated endothelial cells in sinusoid-like vascular structures. In contrast, as shown in Figure 2B, immunohistochemical staining of corresponding tissue sections using a monoclonal antibody against human CD31 was negative, suggesting that ECs in the xenografts were of mouse origin. Similar staining patterns with the two antibodies were observed in corresponding sections of intrahepatic xenografts generated from Huh7 cells (Figure 2C and 2D) and HepG2 cells (Figure 2E and 2F), corroborating the findings in patient-derived xenografts. These observations suggests that intratumoral ECs in human HCC xenografts do not arise from the human TICs but by vessel ingrowth from the surrounding normal murine liver.

To validate this observation directly in primary human HCC tissues, we sought to study patients in whom the origin of intratumoral ECs could be clearly distinguished from surrounding malignant epithelial tissue without complex genetic analyses. Because orthotopic liver transplantation is routinely performed at our institution as definitive therapy for patients with HCC confined to the liver [17], we were able to identify three highly unique patients who had received liver allografts from sex-mismatched organ donors, subsequently developed a new HCC within the transplanted liver, and then had this recurrent HCC resected from the allograft. All of the patients identified were male and, following complete removal of their native livers, had received liver allografts from deceased organ donors who were female. The first patient underwent liver transplantation at age 56 for hepatitis C cirrhosis and a 5 cm moderately differentiated HCC; three years later, he underwent resection of a 9 cm moderately differentiated HCC in the right lobe of the liver allograft. The second patient underwent liver transplantation at age 66 for hepatitis C cirrhosis complicated by moderately differentiated multifocal HCC; he required resection of an 18 cm moderately differentiated HCC in the right lobe of the liver three years after transplantation. The third patient underwent liver transplantation at age 56 for hepatitis B cirrhosis and moderately differentiated multifocal HCC; he underwent resection of a 3 cm moderately differentiated HCC in the left lobe of the liver allograft three years after transplantation.

**Figure 3:**
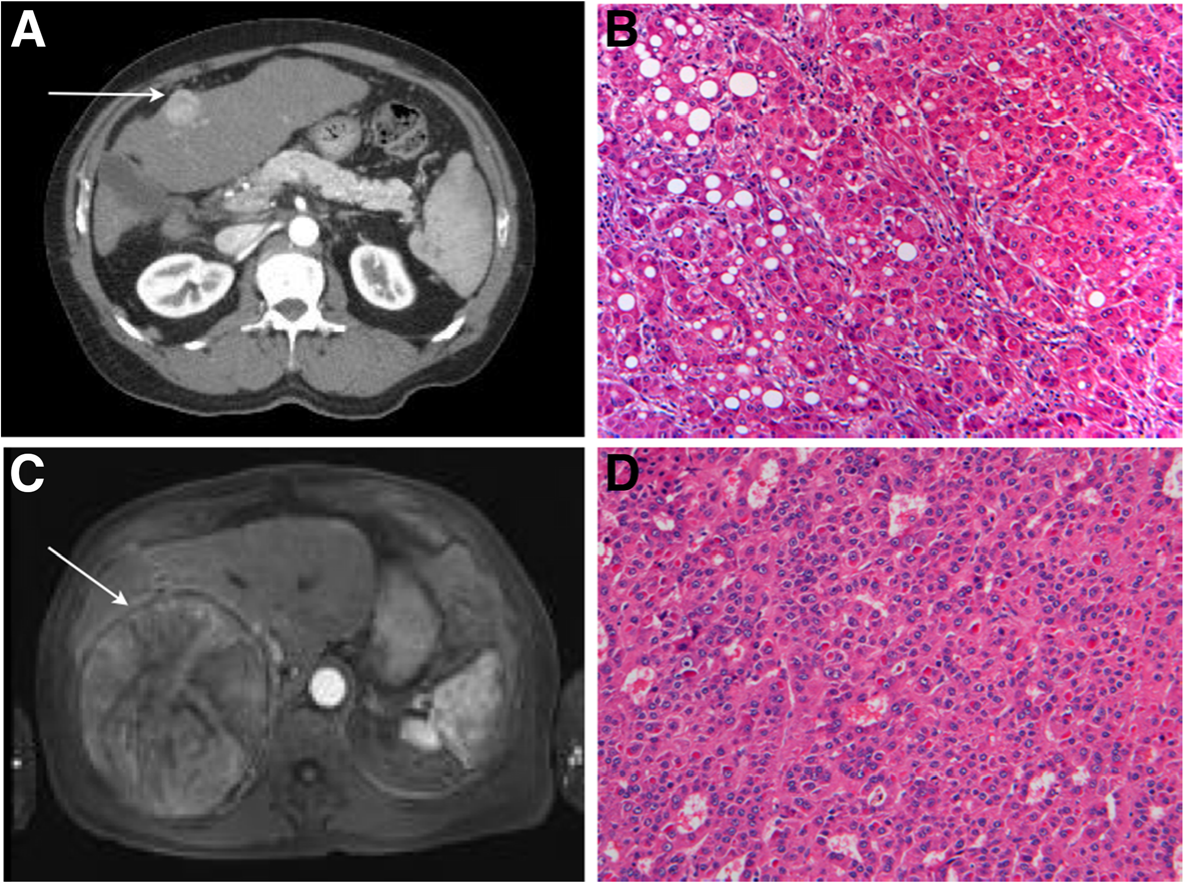
**HCC recurrences in liver allografts of patients who underwent orthotopic liver transplantation for treatment of HCC. A)** Axial computed tomography image demonstrating a hypervascular lesion (arrow) consistent with HCC within a patient’s cirrhotic native liver. **B)** Histopathology of lesion in explanted liver demonstrates features consistent with HCC (H & E stain, x100). **C)** Axial magnetic resonance image of large hypervascular lesion (arrow) within the liver allograft consistent with recurrent HCC. **D)** Histopathology of lesion resected from liver allograft demonstrates features consistent with HCC (H & E stain, x100).

Figure 3 illustrates representative features of the original and recurrent HCCs from one of the patients. The axial computed tomography image in Figure 3A demonstrates a hypervascular lesion consistent with HCC within a patient’s cirrhotic native liver. The histopathology of the lesion, which is consistent with HCC, is shown in Figure 3B. The axial magnetic resonance image in Figure 3C demonstrates a large hypervascular lesion consistent with recurrent HCC within the liver allograft of the same patient. The histopathology of this lesion following resection from the liver allograft is shown in Figure 3D, confirming that it is recurrent HCC. Importantly, the recurrent tumors were discrete lesions within the liver allografts, and not tumors on the liver surface or in the perihepatic tissues that might have resulted from direct tumor seeding into the operative field at the time of transplantation surgery. The appearance of the recurrent HCCs suggests that they either developed as *de novo* malignancies from the liver allograft tissue itself, or from implantation and growth of circulating tumor-initiating cells derived from the patient’s original tumor that persisted in the patient’s lymphatic or vascular system following native total hepatectomy.

**Figure 4:**
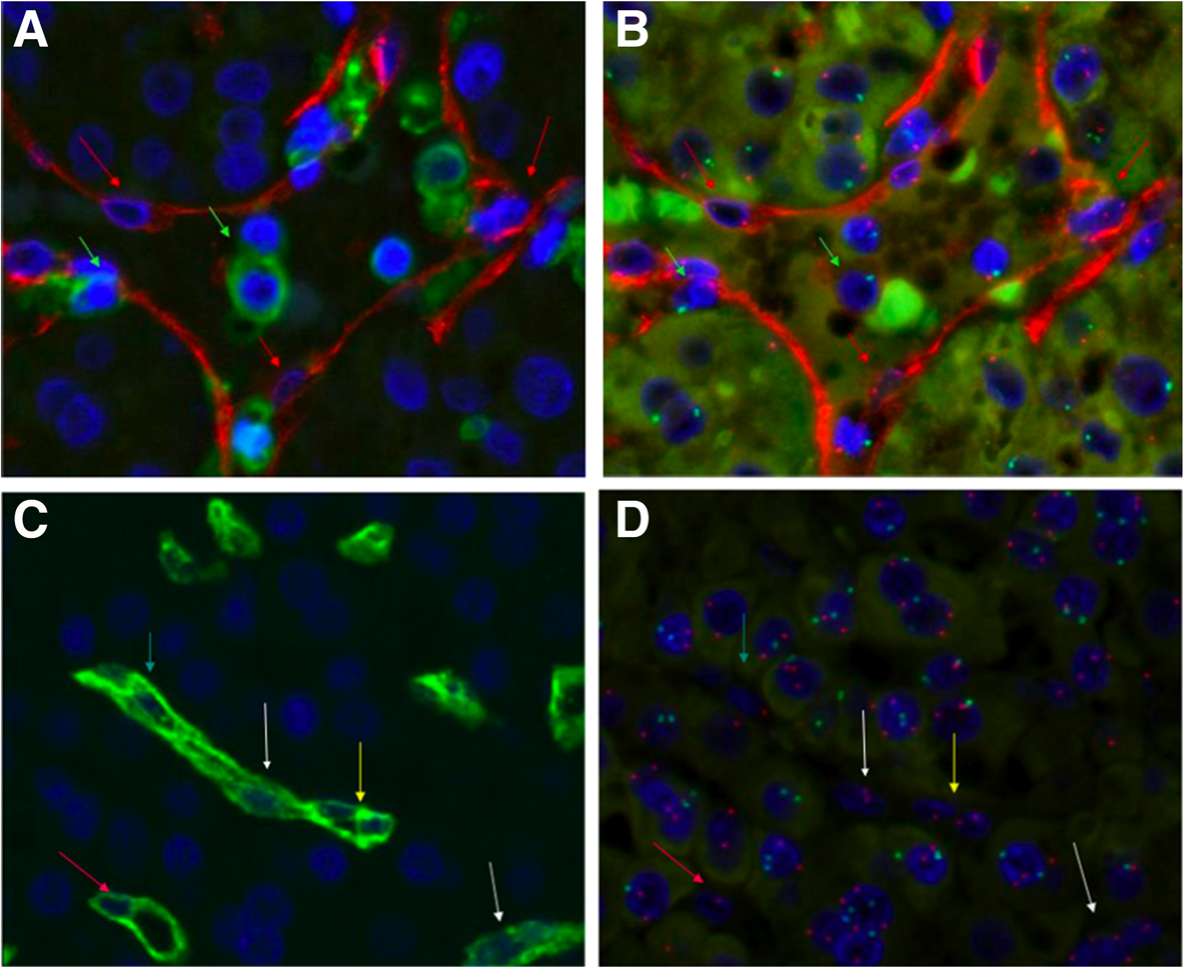
**Endothelial cells within HCC recurrences in patients who received sex-mismatched liver allografts originate from the donor allograft rather than from tumor cells. A)** Representative immunofluorescence staining for CD31 (red) and CD45 (green) identifies ECs (red arrows) and leukocytes (green arrows) within an HCC recurrence that arose in a male patient who received a liver allograft from a female donor (x400). **B)** Fluorescence in situ hybridization (FISH) overlay image of field corresponding to that shown in Panel **A** demonstrating that nuclei of endothelial cells (red arrows) have only X chromosomes (red dots) while leukocytes (green arrows) and tumor cells have both X chromosomes and Y chromosomes (green dots) (x400). **C)** Representative immunofluorescence staining for CD34 (green) identifies sinuisoidal ECs (arrows) within a recurrent HCC (x400). **D)** FISH overlay image of a field corresponding to that shown in Panel **C** demonstrating that endothelial cell nuclei (arrows) have only X chromosomes (red dots) while surrounding tumor cells have both X chromosomes and Y chromosomes (green dots) (x400). The XY phenotype of the tumor cells also confirms that the HCCs occurring in the liver allografts are recurrences of the male patient’s original tumor (XY) rather than malignancies arising from *de novo* transformation of hepatocytes in the XX liver allograft.

Using immunofluorescent staining for CD31 (PECAM-1) and CD34, a marker of sinusoidal ECs in HCC [18], in combination with fluorescence in situ hybridization (FISH) for the X and Y chromosomes, we sought to determine which compartments of the recurrent HCCs had originated from the donor as opposed to the recipient. Figure 4A demonstrates representative immunofluorescence staining for CD31 and CD45 that identifies ECs and leukocytes within an HCC recurrence that arose in a male patient who received a liver allograft from a female donor. Figure 4B shows the FISH overlay image of the corresponding field to that shown in Panel A, illustrating that the nuclei of ECs have only X chromosomes while leukocytes and tumor cells have both X chromosomes and Y chromosomes. Figure 4C demonstrates representative immunofluorescence staining for CD34, which identifies sinusoidal ECs within a recurrent HCC. Figure 4D shows the FISH overlay image of the corresponding field to that shown in Panel C, illustrating that EC nuclei have only X chromosomes while surrounding tumor cells have both X chromosomes and Y chromosomes. Taken together, Figure 4 demonstrates that CD31^+^ and CD34^+^ ECs within the tumor tissue only demonstrate presence of the X chromosome, while the surrounding epithelial cells constituting the bulk of the HCC demonstrate staining for both the X and Y chromosomes. Because the ECs within the recurrent HCCs have an XX phenotype consistent with the female origin of the liver allograft, this suggests that the vascularization of the recurrent HCCs occurred by ingrowth of vessels from the surrounding liver allograft rather than from endothelial differentiation of TICs (in which case the ECs would be XY). In addition, because the tumor cells making up the bulk of the HCCs within the transplanted livers share the sex of the recipients (XY) rather than the donors (XX), this observation suggests that these HCCs were not *de novo* malignancies that arose by transformation of hepatocytes within the allograft (in which case the bulk of the tumor cells would be XX), but rather must have arisen from circulating TICs derived from the recipient’s original HCC.

Limitations of this study include the ability of patient-derived xenograft models to accurately recapitulate all aspects of human cancer pathobiology, which have been criticized due to the immunodeficient state of the murine host and incompatibilities between murine and human factors in the tumor microenvironment [19]. In order to prevent liver allograft rejection, the patients with HCC recurrences that we studied to validate our observations in xenografts were also receiving immunosuppressive therapy, which is known to facilitate cancer development and progression [20]. In addition, all of the resected HCC recurrences that we studied by FISH were moderately differentiated tumors; due to the extremely unique characteristics of the patients from whom these samples originated, we were unable to study specimens that were either well differentiated or poorly differentiated. Although it is possible that endothelial cell origins are different in such tumors, we feel that the observations presented in this study provide valuable insights into the pathobiology of human HCC.

## Conclusions

Taken together, our observations from two unique, biologically relevant, high-fidelity *in vivo* models of functional tumor-initiation strongly suggest that human HCC conforms to conventional models of tumor angiogenesis in which the tumor vasculature is recruited from surrounding “normal” tissues rather than generated through differentiation of TICs. This supports the notion that the biology of TICs may vary between different cancers, and that the observations prominently reported concerning glioblastoma may be specific to that disease. Reports validating a TIC origin of ECs in other cancer types have yet to emerge in the literature. While there is increasing attention to the potential contributions of CSCs/TICs to the pathobiology of human HCC and other tumors, our observations demonstrate that the roles of these cells likely vary between different cancers. The data presented herein firmly support the importance and relevance of many ongoing research efforts targeting pathways and mechanisms of tumor angiogenesis and neovascularization as they are currently understood [21] in order to develop novel therapeutic strategies for this frequently lethal cancer.

## Abbreviations

CSC: Cancer stem cell; EC: Endothelial cell; FISH: Fluorescence *in situ* hybridization; H & E: Hematoxylin and eosin; HCC: Hepatocellular carcinoma; NSG: Non-obese diabetic severe combined immunodeficiency interleukin 2 receptor gamma chain null; RNA: Ribonucleic acid; RT-PCR: Reverse transcription polymerase chain reaction; TIC: Tumor initiating cell.

## Competing interests

The authors declare that they have no competing interests.

## Authors’ contributions

AG conceived of the study, participated in its design and coordination, analyzed the data, and wrote the manuscript. SA and KC carried out the experimental procedures and analyzed the data. OA participated in study design and coordination, data analysis, and preparation of the manuscript. All authors read and approved the final manuscript.

## Pre-publication history

The pre-publication history for this paper can be accessed here:

http://www.biomedcentral.com/1471-2407/13/485/prepub
